# Recovering directed networks in neuroimaging datasets using partially conditioned Granger causality

**DOI:** 10.1186/1471-2202-14-S1-P260

**Published:** 2013-07-08

**Authors:** G Wu, W Liao, S Stramaglia, D Marinazzo

**Affiliations:** 1 Faculty of Psych. and Ed. Sciences, Dept. of Data Analysis, Ghent University, 1, B-9000 Ghent, Belgium; 2 Key Laboratory for NeuroInformation of Ministry of Education, UESTC, Chengdu, 610054, China; 3 Center for Cognition and Brain Disorders, Hangzhou Normal University, Hangzhou 310015, China; 4 Dipartimento di Fisica e Istituto Nazionale di Fisica Nucleare, Sezione di Bari, Bari, 70126, Italy

## 

We address the computational and conceptual problems arising when conditional Granger causality, necessary to disambiguate direct and mediated influences, is used on short and noisy datasets of many variables, as it is typically the case in some EEG protocols and in fMRI. We show that considering Granger causality in the framework of information theory we can limit the conditioning to a limited number of variables chosen as the most informative, obtaining more stable and reliable results on fMRI datasets both at region and voxel level.

The results on a dynamical model simulated on the structural connectome matrix show that PCGC performs significantly better than fully conditioned and pairwise GC, in particular when the signals are short.

We then apply PCGC to publicly available fMRI datasets, with different TRs and divided in two sessions. It is shown that the series with shorter TR have highest informational content. The curve of the mutual information gain when an additional variable is used for conditioning starts to become flat around 10 variables, indicating the optimal number of variables to condition to. These variables are located not only in proximity of the target variable, but distributed across what we could call an "informational resting state network" across the whole brain. This behavior is stable across sections and TRs.

With the additional step of pre-selecting the variables according to the community structure, PCGC is applied as well to time series from individual voxels. This allows to extend the concept of functional connectivity density to effective connectivity, revealing hubs for incoming and outgoing information transfer.

## Conclusions

Apart from solving the computational and conceptual issues arising from a full conditioning of GC, this approach presents a new way of looking at neuroimaging datasets, using the concept of informative clustering to group the variables from different brain regions in terms of their shared information on the future of another target variable.

**Figure 1:**
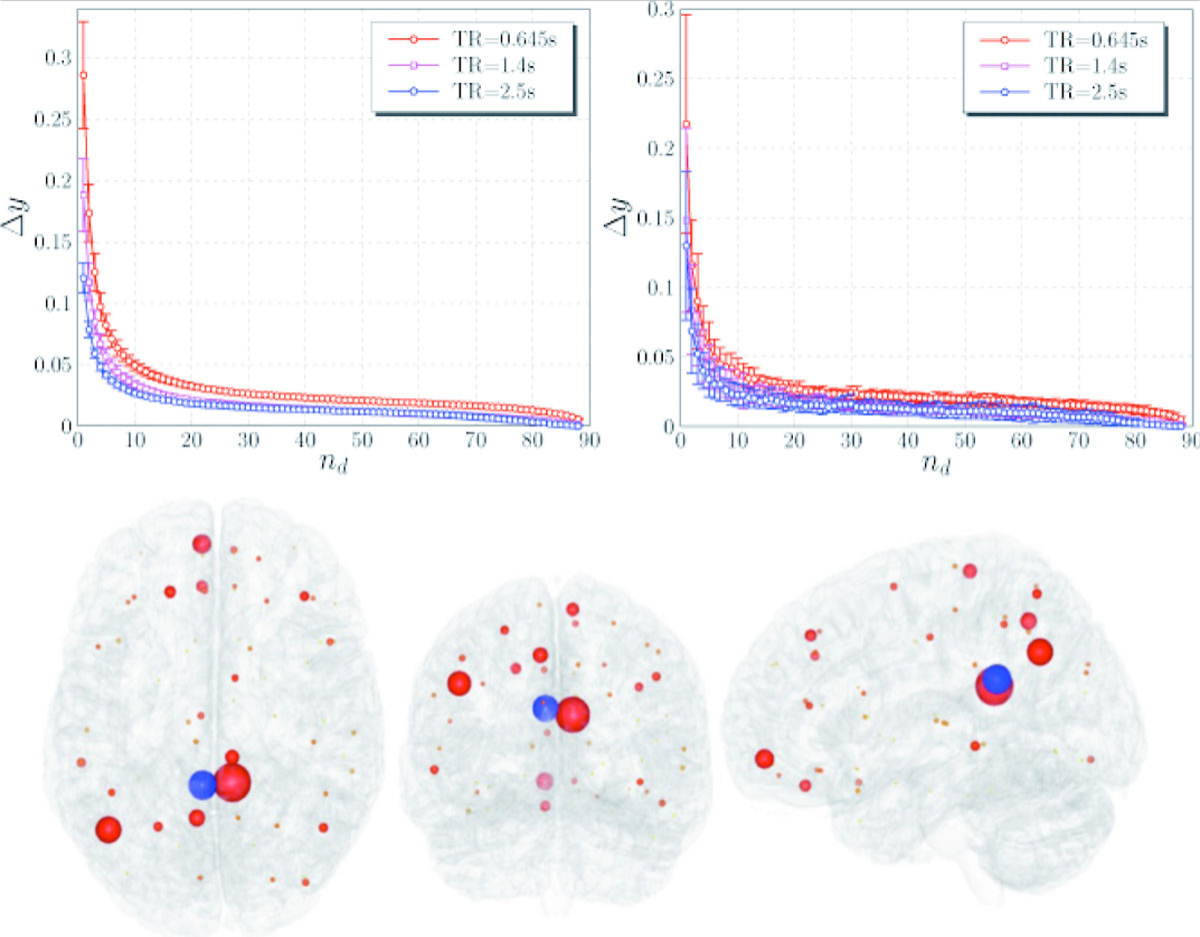
Top: the residual information gain Δy when an additional variable nd is added to the conditioning process, averaged over all targets (left), and for a specific target, PCG.L (right). Bottom: the distributions of the 10 most informative regions for PCG.L

## References

[B1] Deshpande G LaConte S James GA Peltier S Hu X Multivariate Granger causality analysis of fMRI data Hum Brain Mapp 2008 30 1361 1373 18537116 10.1002/hbm.20606 PMC6870657

[B2] Marinazzo D Pellicoro M Stramaglia S Causal information approach to partial conditioning in multivariate data sets Comput Math Methods Med 2012 ID303601 10.1155/2012/303601 PMC3364562 22675400

[B3] Tomasi D Volkow N Functional connectivity density mapping Proceedings of the National Academy of Sciences 2010 107 9885 9890 10.1073/pnas.1001414107 PMC2906909 20457896

